# Collapsibility in case-control studies

**DOI:** 10.1093/ije/dyag085

**Published:** 2026-05-29

**Authors:** Neil Pearce

**Affiliations:** Department of Medical Statistics, London School of Hygiene and Tropical Medicine, London, United Kingdom

**Keywords:** study design, collapsibility, case-control studies

## Abstract

It is well-established that the odds ratio (OR) is non-collapsible and that adjustment of the OR for a risk factor can produce a change in the main effect estimate even when there is no confounding. In contrast, the risk ratio (RR) is collapsible, whereas the rate ratio is non-collapsible, but usually to a lesser extent than is the case with ORs. However, almost all discussions of non-collapsibility have been conducted in the context of effect measures in cohort studies. In this paper, I consider non-collapsibility in case-control studies where the OR inherits the collapsibility or non-collapsibility of the ratio effect measure that the study was designed to estimate. This is most clearly illustrated in the situation of a closed cohort with a fixed follow-up time. In this situation, choosing controls by cumulative incidence sampling estimates the OR in the corresponding cohort study, and therefore the resulting case-control OR is non-collapsible. Choosing controls by case-cohort sampling estimates the cohort study RR, and the resulting case-control OR is collapsible. Choosing controls by density sampling (perhaps the most common approach) estimates the cohort study rate ratio, and the resulting case-control OR is non-collapsible, but to a lesser extent than for cumulative incidence sampling. Moreover, if the disease under study is rare (in the cohort study), all three methods produce case-control ORs that are approximately collapsible.

Key Messages

- The odds ratio (OR) is non-collapsible, and adjustment of the OR for a potential confounder in a cohort study can produce a change in the main effect estimate even when there is no confounding.
- The OR in a case-control study inherits the collapsibility or non-collapsibility of the ratio effect measure that the study was designed to estimate
- In case-control studies, whether the case-control OR is collapsible depends on how the controls were chosen: case-cohort sampling produces ORs that are collapsible, whereas cumulative incidence sampling or density sampling produces ORs that are not collapsible.
- If the disease under study is rare (in the cohort study), all three standard methods for choosing controls in the corresponding case-control study produce case-control ORs that are approximately collapsible.

Non-collapsibility has been defined as a ‘failure of a measure when taken as a group to equal a simple average of the measure when taken on the group’s members or subgroups’ [1]. This particularly occurs when the odds or odds ratio (OR) is the outcome measure of interest in an epidemiological study [2]. It also occurs, but usually to a lesser extent (the reason for this is explained below), when the incidence rate or rate ratio is the outcome of interest. It does not occur when the risk or RR is the outcome of interest [3]. Similar concerns apply when using these three effect measures to estimate absolute effect measures (which can be done directly in a cohort study and indirectly in a case-control study) that matter for public health. However, non-collapsibility is of only trivial concern when a rare disease is being studied, since in this situation, the OR, rate ratio, and RR are all approximately equal.

In fact, most of the published examples and simulations of non-collapsibility involve relatively extreme examples, where the odds are substantially greater than 1.0 in at least one subgroup, and the OR is therefore markedly different from the corresponding RR [4]. One might argue that such situations rarely occur in epidemiological practice, since odds greater than 1.0 means that more than one-half of the study participants have experienced the outcome. Nevertheless, it can still be of potential concern in some (perhaps unusual) situations [5], particularly since the odds may be relatively large in some subgroups, even if they are not large overall, when there is adjustment for multiple potential confounders [3].

The problem of non-collapsibility is of particular concern for modelling strategies, because it complicates the assessment of confounding. In particular, the ‘change-in-estimate’ (CIE) approach is often used in epidemiology to assess potential confounders [6, 7] and is a very convenient approach in many standard data analyses (in fact, most authors do not say anything about what modelling strategy they have used, but in practice it is usually a variant of one of these approaches: (i) use of a model with all measured covariates *a priori* identified as potential confounders, (ii) testing of covariate coefficients to eliminate some variables from a model predicting outcome or exposure (as in stepwise regression), or (iii) using a CIE criterion to eliminate variables [6, 7]). However, the CIE approach can be misleading if there is a problem of non-collapsibility, since in this situation, adjustment for a risk factor can produce a change in the main effect estimate even when there is no confounding [8]. The strength of this non-collapsibility effect (defined as the difference between the marginal and conditional exposure-outcome associations) also depends on the prevalence of the relevant risk factor that is being stratified [9]. One solution to this problem is to use standard OR methods, such as logistic regression (which provides ease of statistical manipulation and estimation [10]), and to then construct collapsible measures, such as covariate-specific and weighted-average (standardized) RRs [3]. However, these methods are currently not used widely, perhaps because of their computational complexity (although they are less complex than some other methods such as G-computation or machine learning that are becoming more widely used).

Almost all discussions of non-collapsibility have been conducted in the context of effect measures in cohort studies. The motivation for this paper is that, in my experience, many colleagues take the finding that ‘the OR is not collapsible’ to also apply to ORs in case-control studies. In this paper, I therefore consider non-collapsibility in case-control studies and show that whether or not the case-control OR is collapsible depends on how the controls were chosen, i.e. the OR in a case-control study inherits the collapsibility or non-collapsibility of the ratio effect measure that the study was designed to estimate. This is most clearly illustrated in the situation of a closed cohort with a fixed follow-up time. I start by considering the relevant issues in such a cohort study before proceeding to consider the corresponding issues in case-control studies based on such a cohort study.

## What happens in a cohort study?

The problem of non-collapsibility is perhaps most relevant when the CIE approach might result in a decision to adjust for a potential confounder (because the adjusted estimate is different from the unadjusted estimate) even when there is in reality no confounding (the situation where the CIE approach incorrectly leads to the conclusion that there is no confounding when there actually is, is of less concern, because in this situation the adjusted and non-adjusted effect estimates are the same, so the decision to adjust or not to adjust has little or no effect on the results). This situation of ‘no confounding’ is illustrated in Table 1, which shows the results of a hypothetical study in a closed cohort involving 20 000 exposed and 20 000 non-exposed persons, followed for up to 10 years. For simplicity, I assume that we are studying disease incidence and that there are no relevant mediators or colliders and, therefore, that confounding is the main issue of concern. In Table 1, equal numbers of males and females are in the study, and equal numbers of each (10 000) are exposed. Thus, there is no confounding by gender. The table also shows the three main relative effect measures that can be used in such a cohort study: (i) the odds, i.e. the ratio of cases to non-cases, (ii) the risk, i.e. the ratio of cases to the total population at baseline, and (iii) the incidence rate, i.e. the ratio of cases to person-years. I have assumed, for the purposes of this illustration, that no one is lost to follow-up and that the cases, on average, occur halfway through the 10-year follow-up period, so cases each contribute 5 years of follow-up (before they become a case) and non-cases contribute the full 10 years of follow-up.

**Table 1:** Effect estimation and non-collapsibility for the odds ratio, risk ratio, and incidence rate ratio.

Cohort study										
	Males			Females			Total			Adjusted
	Exposed	Non-exp	Total	Exposed	Non-exp	Total	Exposed	Non-exp	Total	
**Cases**	4000	2000	6000	2000	1000	3000	6000	3000	9000	
**Non-cases**	6000	8000	14 000	8000	9000	17 000	14 000	17 000	31 000	
**Total**	10 000	10 000	20 000	10 000	10 000	20 000	20 000	20 000	40 000	
**Pyrs**	80 000	90 000	170 000	90 000	95 000	185 000	170 000	185 000	35 5000	
**OR** ^a^	2.67			2.25			2.43			2.50
**RR** ^b^	2.00			2.00			2.00			2.00
**IRR** ^c^	2.25			2.11			2.18			2.20

Case-control study										
	Males			Females			Total			Adjusted
	Exposed	Non-exp		Exposed	Non-exp		Exposed	Non-exp	Total	
**Cases**	4000	2000	6000	2000	1000	3000	6000	3000	9000	
**CI** ^a^	1742	2323	4065	2323	2613	4935	4065	4935	9000	
**CC** ^b^	2250	2250	4500	2250	2250	4500	4500	4500	9000	
**Density** ^c^	2028	2282	4310	2282	2408	4690	4310	4690	9000	
**OR_OR** ^a^	2.67			2.25			2.43			2.50
**OR_RR** ^b^	2.00			2.00			2.00			2.00
**OR_IRR** ^a^ ^,^ ^c^	2.25			2.11			2.18			2.20

a OR, odds ratio; controls chosen by cumulative incidence (CI) sampling to estimate the OR from the cohort study.

b RR, risk ratio; controls chosen by case-cohort (CC) sampling to estimate the RR from the cohort study.

c IRR, incidence rate ratio; controls chosen by density sampling to estimate the IRR from the cohort study.

The table also shows that the exposed group has twice the disease incidence proportion of the non-exposed group, so that the RR is 2.0 overall and is also 2.0 in males and 2.0 in females. Thus, the crude (unadjusted) RR is a simple average of the gender-specific RRs and is equal to the Mantel–Haenszel-adjusted RR. Given that there is an effect of exposure, but there is no effect modification by sex when using the RR, there will inevitably be effect modification by sex when using the OR or incidence rate ratio (IRR). However, the effect modification in this example is small, and the issue of effect modification is beyond the scope of this paper (in any case, even if effect modification exists, it is still reasonable to estimate the ‘average population effect’).

The situation is different for the OR. This is 2.67 in males and 2.25 in females, and the crude overall OR is 2.43, which is not a simple average of the two. Also, the adjusted OR is 2.50, which is slightly different from the crude OR, even though there is no confounding. This occurs because the exposed group has more events (cases) than the non-exposed group, and males have more events than females. This means that the ‘non-cases’ (the denominators used to calculate the ORs) are not evenly distributed (there are more non-cases among the non-exposed and among females) at the end of follow-up (even though they were evenly distributed at the start of follow-up). This produces an imbalance in the denominators used to calculate ORs (i.e. the non-cases) and, hence, the phenomena of non-collapsibility.

The table also shows that the same phenomena occur with the IRR, but to a much smaller extent in this example—the crude rate ratio is 2.18, compared with a Mantel–Haenszel adjusted estimate of 2.20. Why is the problem of non-collapsibility of lesser strength when using the IRR? The table shows that when the number of incident cases is affected, the number of non-cases and the person-time at risk are affected as well. However, if a case occurs, this means that a non-case is ‘eliminated’ (it changes into a case) from the denominator for the odds. In contrast, the corresponding person-time of the incident case is not completely eliminated from the denominator for the rate because the non-case still contributes some person-time before it becomes a case (i.e. not all of its person-time is ‘lost’ to the denominator—in this example, I have assumed that only half of it is lost). In contrast, the denominator for the RR remains the same, no matter how many incident cases occur.

Thus, non-collapsibility of the OR and rate ratio can occur, indicating a difference between the adjusted and unadjusted effect estimates, even when there is no confounding. It occurs because the fact that the exposed group, and males, experience more events and therefore ‘lose’ more non-cases and more person-years. For the risk, if an event occurs, then this adds ‘1’ to the numerator but does not affect the denominator (since this is always the same as the numbers at the start of follow-up). For the odds, if an event occurs, then this adds ‘1’ to the numerator and subtracts ‘1’ from the denominator; hence, the problem of non-collapsibility. For the rate, if an event occurs, then this adds ‘1’ to the numerator, but the number of person-years that are ‘removed’ from the denominators depends on when the event occurs—if the event occurs on the last day of follow-up, then there is no change in the denominator and there is no problem of non-collapsibility; if the event occurs on the first day of follow-up, then all of the corresponding person-years are ‘removed’ and the problem is as large as the situation for the odds (where the relevant person is completely removed from the denominator). Thus, the denominators used to estimate the odds (cases) and the rate (person-years) are associated with exposure status and with gender, in the denominators for odds and rate analyses, even though they are not associated with exposure in the denominators for risk analyses. These latter denominators represent the study population at baseline and are therefore the reference for assessing potential confounding.

In fact, the problem of non-collapsibility in this example is relatively trivial—there is little difference between the adjusted and non-adjusted OR and rate ratio estimates. This occurs even though the example uses a relatively common outcome—overall, 22.5% of participants experience the outcome. As noted above, non-collapsibility is only a problem in practice in relatively extreme situations where the odds are very large in at least some subgroups. Nevertheless, it is a potential problem that needs to be considered when using the CIE approach to the assessment of confounding.

## What happens in case-control studies?

So what is the situation in case-control studies? My experience has been that various colleagues often assume that non-collapsibility is a major problem in this situation because case-control studies involve the estimation of ORs, and these usually involve approximately equal numbers of cases and non-cases (i.e. the odds are often about 1.0). However, it is well-known and well-established that what the OR estimates in a case-control study depends on how the controls were chosen [11]. In particular, there are three main methods of control selection in a case-control study, which correspond to the three main methods of analysis of the corresponding cohort study: (i) cumulative incidence sampling involves selecting controls from the non-cases, i.e. the denominators used to estimate the odds in a cohort study, (ii) case-cohort sampling involves selecting controls from the entire study population (at baseline), i.e. the denominators used to estimate the risk in a cohort study, and (iii) density sampling involves selecting controls from the person-years, i.e. the denominators used to estimate the incidence rate in a cohort study.

The lower half of Table 1 shows the findings of three case-control studies created by these three methods of sampling. For example, there are 9000 cases in the cohort, so choosing controls at random from the study population (case-cohort sampling) results (on average) in equal numbers of controls (2250) in each of the four groups (exposed/non-exposed, males/females), and the resulting case-control OR (2.00) exactly estimates the corresponding cohort RR. Also, just as in the cohort study, there is no problem of non-collapsibility. Similarly, choosing controls by cumulative incidence sampling produces a case-control study where the OR exactly estimates the corresponding OR in the cohort study, and there is a (small) problem of non-collapsibility, just as in the cohort study. Finally, choosing controls by density sampling exactly estimates the corresponding rate ratio in the cohort study, and there is a (smaller) problem of non-collapsibility, just as in the cohort study. Thus, unsurprisingly, the problem of non-collapsibility is exactly the same (and just as trivial) in the three case-control studies as it is in the full cohort study.

So what does this mean in practice? It means that we cannot make general statements about the collapsibility of the OR in a case-control study unless we know how the controls were chosen. It also means that for a rare disease, non-collapsibility of the OR is not a problem in a case-control study (even though the odds may be large in the case-control study), just as it is not a serious problem in the corresponding cohort study. And for those rare instances where we are studying a very common outcome (much more common than that in Table 1), non-collapsibility of the OR in a case-control study is still not a problem if the controls were chosen by case-cohort sampling and may be a relatively small problem if controls were chosen by density sampling. Fortunately, the latter approach (i.e. density sampling) is the norm in most modern case-control studies [11], whereas cumulative incidence sampling is rarely used nowadays, just as ORs are rarely used for analysing cohort studies.

In summary, in case-control studies, whether the case-control OR is collapsible depends on how the controls were chosen: case-cohort sampling produces ORs that are collapsible, whereas cumulative incidence sampling or density sampling produces ORs that are not collapsible. However, if the disease under study is rare (in the corresponding cohort study), all three standard methods for choosing controls in the corresponding case-control study produce case-control ORs that are approximately collapsible, and the problem of non-collapsibility is relatively trivial.

## Ethics approval

Not applicable.

## Artificial intelligence

Not used.
